# Sensory Amplitude Electrical Stimulation via Sock Combined With Standing and Mobility Activities Improves Walking Speed in Individuals With Chronic Stroke: A Pilot Study

**DOI:** 10.3389/fnins.2019.00337

**Published:** 2019-04-16

**Authors:** Roberto Lopez-Rosado, Andrea Kimalat, Matthew Bednarczyk, Jane E. Sullivan

**Affiliations:** ^1^Physical Therapy & Human Movement Sciences, Feinberg School of Medicine, Northwestern University, Chicago, IL, United States; ^2^Northwestern Memorial Hospital, Chicago, IL, United States

**Keywords:** stroke, rehabilitation, lower limb, electrical stimulation, gait, function

## Abstract

**Objective:** To determine if sensory amplitude electrical stimulation (SES) delivered via sock electrode combined with standing and mobility activities improved gait speed, sensation, balance, and participation in chronic stroke. It was hypothesized that SES would enhance the effectiveness of exercise, resulting in reduced impairment and improved function.

**Design:** Case Series.

**Setting:** Home-based intervention.

**Participants:** Thirteen adults (56.5 + 7.84 years old) with chronic stroke (8.21 + 4.36 years post) and hemiparesis completed the study. Participants were community ambulators.

**Intervention:** Participants completed 6 weeks of self-administered SES delivered via sock electrode concurrent with standing and mobility activities for a minimum of 5 days/week for 30-min, twice daily.

**Outcome Measures:** Berg Balance Scale (BBS), Stroke Rehabilitation Assessment of Movement—LE subscale (STREAM), 10 Meter Walk Test (10 MWT), Activities-Specific Balance Confidence Scale (ABC), Stroke Impact Scale (SIS), Perceptual Threshold of Electrical Stimulation (PTTES), and Monofilament testing were administered at pre-test, post-test, and 3-month follow up.

**Results:** Baseline sensory scores and change scores on functional outcomes were analyzed using Pearson Product-Movement Correlation Coefficients, Friedman test, and Linear mixed models. There was a significant change with 10 MWT self-selected pace (Friedman's *p* = 0.038). Pre-post intervention changes in other outcome measures were not significant. According to the Cohen's effect size classification, there were medium effect sizes for both the STREAM-LE and Monofilaments.

**Conclusion:** The use of home-based SES via sock electrode combined with standing and mobility activities may contribute to improve gait speed in chronic stroke.

## Introduction

Stroke is a leading cause of disability and the fifth leading cause of death in adults in the United States (Mozaffarian et al., 2015). Approximately 50% of this population regains independent ambulation by the end of rehabilitation; however 73% have some degree of long-term gait disability (Woolley, 2001). The amount of community walking done by individuals post-stroke is considerably less than their healthy peers (Michael and Macko, 2007). Falls are a serious consequence of stroke (Langhorne et al., 2000), with more than half of individuals experiencing a fall (Ashburn et al., 2008; Sackley et al., 2008). Post stroke falling is associated with gait and balance dysfunction (Minet et al., 2015). Post stroke changes in sensory dysfunction (Carey, 1995; Winward et al., 2007; Connell et al., 2008; Tyson et al., 2008) have also been associated with falls following stroke (Yates et al., 2002; Tyson et al., 2006; Wutzkea et al., 2013).

Sensory amplitude stimulation (SES) is electrical stimulation utilized at a threshold to stimulate sensory neurons only without stimulating motor neurons. In healthy adults, SES has been reported to enhance cortical motor excitability (Hamdy et al., 1998; Ridding et al., 2000; Kaelin-Lang et al., 2002; Golaszewski et al., 2004; Tinazzi et al., 2005; Meesen et al., 2010) and produce short-term plastic changes in the motor and sensory cerebral cortices (Ridding et al., 2001; Tinazzi et al., 2005; Wu et al., 2005). The enhanced afferent input provided by SES has been hypothesized to contribute to motor recovery of individuals with neurological conditions (Kwong et al., 2008; Bastos Conforto et al., 2018).

In adults post-stroke, SES use has been associated with body structure/function-domain improvements in force (Ng and Hui-Chan, 2007; Klaiput and Kitisomprayoonkul, 2009; Yan et al., 2009; Conforto et al., 2010; Tyson et al., 2013) and sensation (Ng and Hui-Chan, 2007; Tyson et al., 2013). Positive activity-domain changes such as improved gait speed, walking distance, balance, trunk control, and foot placement have been reported. Use-dependent plastic changes in the sensorimotor cortex following SES intervention have been demonstrated post-stroke. A recent systematic review on SES use following stroke examined 15 studies and concluded that the intervention has beneficial effects on motor recovery, especially when concurrently administered with standing and mobility activities The diverse outcome measures used across studies precluded meta-analysis, but the majority of studies reported significant effects on at least one outcome.

While preliminary positive findings following SES interventions post-stroke have been reported, important gaps in the literature remain. Only a small number of studies have examined retention by conducting follow up testing. The majority of SES studies to date have utilized surface electrodes located either over muscles or nerves corresponding to the target function. This electrode placement might actually interfere with activity. Several recent studies have utilized wearable electrodes (socks, gloves), which enable peripheral stimulation over a broad receptor field concurrent with activity (Ng and Hui-Chan, 2007; Yan et al., 2009; Tyson et al., 2013). Finally, studies have been primarily conducted in a clinic or research lab. Few studies have sought to examine whether beneficial effects can occur with a home-based SES intervention.

The aim of this study was to examine the effects of a 6-week intervention of home-based SES via sock electrode delivered during standing and mobility activities in individuals with chronic stroke. This will be known as “SES plus activity.” Our primary hypothesis was that there would be significant improvements in gait speed, balance, and balance confidence, which would be retained at 3-months post-intervention. We also hypothesized that there would be improvements in quality of life and in lower extremity active movement.

## Methods

### Participants

Participants were recruited from a university-based stroke database. Inclusion criteria were: (1) diagnosis of chronic stroke (>6 months); (2) able to ambulate in the community; (3) at least 21 years of age; (4) English-speaking. Exclusion criteria were: (1) contraindications to electrical stimulation (such as active local infection, inflammation, or malignancy); (2) positive history of neurologic diagnosis other than stroke; (3) chemodenervation (e.g., *Botox*^TM^) in the more involved lower extremity within the past 6 months. All participants were informed of their rights and the expectations for the study. Participants provided informed consent per the protocol as outlined by the Northwestern University Institutional Review Board Office.

### Experimental Design

This study used a case series design with a pre-test, post-test, and follow-up. All assessment sessions were completed in a university setting. Research participants completed the 6-week intervention primarily in their home and community. We used STROBE cohort reporting guides (von Elm et al., 2007).

### Intervention

Electrical stimulation for SES plus activity was delivered using a muscle stimulator (EMPI, Inc., St. Paul, MN, USA) through a Silver-Thera sock electrode (Prizm Medical, Inc., Duluth, GA, USA) worn on the more involved foot. A secondary 2 × 2″ pre-gelled electrode was placed over the *Tibialis anterior* muscle belly. Stimulator parameters were as follows: symmetrical biphasic waveform, pulse duration 250 ms, stimulus frequency 50 Hz, duty cycle 10-s ON: 10-s OFF, and amplitude above sensory threshold but below motor threshold. Participants performed standing and mobility activities while receiving SES plus activity for a minimum of 30 min twice a day, 5 days/week for 6 weeks. Participants were not provided with a standard set of upright activities. They were only instructed to be standing and active during the stimulation. Participants reported walking around their home, leaving the house to run errands to the pharmacy and supermarket, attending church services and doctor's appointments. A compliance meter on the stimulator captured stimulation time, but didn't account for movement time. Additionally, all participants completed a daily log sheet and recorded stimulation time and standing and mobility activities. Daily logs were detailed and discussed with all participants during reassessment times; this method proved to be efficacious as our participants reported feeling independent while being accountable recording their activities.

Participants returned to the university setting for a minimum two sessions during the 6-week period. The purpose of the visits was to monitor adherence, answer participant questions, to assess the stimulator, and readjust the sensory threshold if needed.

### Outcome Measures

The outcome measures administered at pre-test, post-test, and 3 months follow-up included:

#### Primary Measures

##### 10 meter walk test (10MWT)

The reliability and responsiveness of this test has been established in chronic stroke, as well as its high correlation with self- reported outcome measures in this population. Lower scores correspond to higher gait speed and lower risk for falls. Both self-selected and fast pace were assessed (Bushnell et al., 2015).

##### Activities of balance confidence scale (ABC)

This is a self-reported survey that captures participant's perceived balance confidence in a 0–100% range (the higher the score, the better the perceived balance). Reliability and normative scores have been reported for chronic stroke (Salbach et al., 2006).

##### Berg balance scale (BBS)

The BBS examines static and dynamic balance performance. Reliability and responsiveness data is highly correlated with the ABC for individuals post-chronic stroke. Higher scores are indicative of better balance performance (Salbach et al., 2006).

### Secondary Measures

#### Stroke impact scale (SIS)

This is a multi-dimensional, self-reported health status measure post-stroke. Reliability and validity has been established specifically for individuals post-stroke. Responsiveness and normative data have been reported (Bushnell et al., 2015).

#### Stroke rehabilitation assessment of movement- lower extremity subscale (STREAM-LE)

The STREAM-LE was used to assess voluntary movement in the lower extremity. It has established reliability and responsiveness and normative data in adults post-stroke, and it is recommended as one assessment for function and strength in the paretic lower extremity (Huang et al., 2015).

#### Monofilaments

Assessment with monofilaments was conducted with the Semmes-Weinstein monofilaments (SWM) test on the sole of the paretic feet. Reliability of the SWM test was high on the paralyzed side (rs = 0.86, κ = 0.71–0.79), it was low on the other side without paralysis (rs = 0.33–0.50, κ = 0.33–0.50; Arakawa et al., 2012).

### Statistical Analysis

Friedman tests were used to assess overall change over time. Linear mixed models were used as post-tests to demonstrate between which assessment points a significant change was seen. Due to the small sample size, these models were also fit adjusting for one covariate at a time.

Baseline sensory scores and change scores on functional outcomes were analyzed using Pearson Product-Movement Correlation Coefficients. Effect sizes were calculated for each of the outcome measures, as previous similar studies (Sullivan et al., 2015).

## Results

Fifteen participants were enrolled; 13 completed the study. Table 1 summarizes participants' characteristics. Two participants dropped^*^ from the study because of personal reasons unrelated to the study.

**Table 1:** Participant demographic and baseline data.

**ID**	**Age (years)**	**Sex**	**Years since stroke**	**Body side involved**	**Baseline scores**					
					**10 MWT (m/s) self-selected**	**10 MWT (m/s) fast**	**ABC (%)**	**BBS (/56)**	**STREAM LE (/20)**	**Mono filaments**
1	65	M	9	L	0.97	2.63	83.75	55	9	4.56
2	47	M	15	L	0.63	0.74	81.88	50	10	7.80
3	45	M	10	R	0.83	1.26	72.50	56	9	4.31
4^*^	67	M	8	L	0.68	0.81	78.13	51	11	4.0
5	44	M	4	R	0.87	1.27	68.75	56	12	7.8
6	60	M	6	R	0.82	1.17	85.63	55	9	4.31
7^*^	62	M	5	R	0.66	1.11	80	53	15	4.31
8	53	M	10	R	0.53	0.84	73.13	46	11	4.56
9	49	M	16	R	0.88	1.44	85.63	52	11	4.56
10	63	M	7	L	0.22	0.24	66.88	32	7	6.65
11	56	F	6.5	L	0.76	1.62	77.19	51	10	5.07
12	60	M	0.75	R	0.78	1.07	95	56	8	4.56
13	62	F	7	L	0.65	0.75	71.56	53	8	4.31
14	55	M	13.5	R	0.31	0.5	65	42	8	6.65
15	61	M	7	L	0.54	0.89	46.56	53	8	6.65
Median	60		7		0.68	1.07	77.19	53	9	4.56
Interquantile range	13		4		0.29	0.52	15	5	3	2.34

*Participant demographic and baseline data. M, male; F, female; L, left; R, right; 10 MWT, 10 Meter Walk Test; S, Self-Selected Pace; F, Fast Pace; ABC, Activities-Specific Balance Confidence Scale; BBS, Berg Balance Scale; STREAM LE, Stroke Rehabilitation Assessment of Movement, leg subscale*.

* *Individuals who dropped the study*.

Table 2 illustrates the mean outcome measures over time of those who completed the study. There was a significant change over time with 10 MWT at self-selected pace (Friedman's *p* = 0.038). Using a linear mixed models analysis, there was a significant effect on 10 MWT at self-selected pace, *p* = 0.030, comparing baseline to post-test. This remained significant after adjusting for time from onset of stroke, or the use of an assistive device. The change in other outcome measures between assessment periods was not significant.

**Table 2:** Mean (SD) of outcomes measures over time.

	**Baseline**	**Post**	**Follow Up**	**Friedman's *p***
10 MWT (m/s) S^*^	0.69 (0.22)	0.78 (0.25)	0.76 (0.26)	**0.038**
10 MWT (m/s) F^*^	1.05 (0.49)	1.11 (0.44)	1.07 (0.45)	0.066
ABC (%)	75.4 (11.4)	74.3 (18.2)	73.7 (14.7)	0.926
BBS (/56)	50.7 (6.5)	51.1 (7.4)	50.8 (8.1)	0.911
SIS	3.88 (0.39)	3.84 (0.33)	3.76 (0.29)	0.458
STREAM-LE (/20)	9.73 (2.05)	9.92 (1.49)	9.77 (2.12)	0.226
Monofilaments	5.34 (1.357)	5.36 (1.532)	5.38 (1.519)	0.310

* *S-self-selected pace; F-fast pace. 10 MWT, 10 Meter Walk Test; S, Self-Selected Pace; F, Fast Pace; ABC, Activities-Specific Balance Confidence Scale; BBS, Berg Balance Scale; SIS, Stroke Impact Scale; STREAM LE, Stroke Rehabilitation Assessment of Movement, leg subscale. Bold value indicates the level of significance*.

To account for sample size, Cohen's effect size classifications were determined (Cohen, 1988). There were medium effect sizes for both the STREAM-LE and Monofilaments in participants from baseline to follow up. A large effect size was also observed for both 10 MWT at self-selected and fast pace (see Table 3).

**Table 3:** Effect size of treatment between Baseline and Follow-Up.

	**Baseline → follow up**
STREAM-LE	0.371
Monofilaments	0.455
10 MWT (m/s) *S*, 1st trial	0.689
10 MWT (m/s) *F*, 1st trial	0.568

Baseline sensory status of the hemiparetic plantar surface of the foot was assessed via the Perceptual Threshold of Electrical Stimulation (PTTES) and Monofilament testing on three locations. This tool has been shown to be reliable for testing sensation following stroke.

Baseline sensory status (both PTTES & Monofilaments) showed moderate to strong correlation with Baseline SIS 16 scores and change scores on the SIS 16 and ABC, suggesting sensory status may be associated with self- perception of physical performance (refer to Table 4).

**Table 4:** Correlations between outcome measures and sensory data.

**Outcome measures and mean scores**		**Baseline PTTES**			**Baseline monofilaments**		
		**Baseline**	**Baseline to post Δ**	**Baseline to F/U Δ**	**Baseline**	**Baseline to post Δ**	**Baseline to F/U Δ**
10-Meter Walk Test (10 MWT) with Self-Selected (SSP) and Fast Pace (FP)	SSP: 0.7 m/s (0.28–1.12)	0.278	0.121	0.087	0.340	0.244	0.391
	FP: 1.02 m/s (0.46–1.81)	0.168	0.056	0.121	0.360	0.179	0.383
Activities-Specific Balance Confidence Scale (ABC)	$\bar{x}$: 75.4% (46.5–95/100)	0.183	0.535	0.777^**^	0.439	0.292	0.044
Stroke Impact Scale (SIS)	$\bar{x}$: 65% (50–85%)	0.679^**^	0.278	0.222	0.209	0.091	0.137
*SIS 16^*^a^*^*	$\bar{x}$: 82%	0.824^**^	0.631^*^	0.560^*^	0.052	0.065	0.076
*SIS mobility^*^b^*^*	$\bar{x}$: 82%	0.715^**^	0.526	0.538	0.069	0.151	0.013
*SIS participation^*^c^*^*	$\bar{x}$: 71%	0.359	0.063	0.270	0.649^**^	0.425	0.314
Berg Balance Scale (BBS)	$\bar{x}$: 50/56 (32–56/56)	0.167	0.138	0.266	0.274	0.033	0.147
Stroke Rehabilitation Assessment of Movement (STREAM)	LE Subscale: $\bar{x}$: 9.67/20 (7–14/20)	0.123	0.065	0.316	0.123	0.459	0.429
**Correlations** (Explorable.com and Wilson, 2009)							
Weak 0.2–0.29	Moderate 0.3–0.39		Strong 0.4–0.69		Significant ^*^p ≤ 0.05		Significant ^**^p ≤ 0.01

a *SIS 16: a subset of 16 items capturing daily activities from the SIS*.

b *SIS mobility: a subset of 9 items capturing mobility items from the SIS*.

c *SIS participation: a subset of 8 items capturing participation and role function from the SIS*.

## Discussion

Participants experienced significant group effects changes in self-selected walking speed following SES plus activity in the home and community setting. These effects were maintained after the end of the intervention, but they weren't sustained at follow up.

Moderate to strong correlations with Baseline SIS 16 scores and change scores on the SIS 16 and ABC may suggest that sensory status may be associated with self- perception of physical performance. Greater sensory impairment was associated with lower self- reported baseline status. It remains unclear whether utilizing PTTES and/or Monofilaments to assess sensory status is the optimal approach.

The significant improvement in the gait speed of individuals with a more recent stroke and younger age may be related to an enhanced neural plasticity potential. The medium effect sizes in the STREAM-LE supports that possibility. Stimulating the paretic distal lower extremity at the sensory level may have an effect on the motor output level required for self-selected gait speed, suggesting a sensorimotor integration loop. This may potentially influence optimal SES dosage parameters to positively affect self-selected gait speed, which has implications on balance functional levels in individuals post-stroke at a chronic stage.

There were no other changes in other administered outcome measures. Participants scored highly at baseline on the BBS and ABC, so there might have been a ceiling effect. Alternatively, these outcome measures may have been less sensitive to changes with this intervention.

The sock electrode delivered electrical stimulation over the entire surface of the foot and distal leg instead of targeting specific distal lower extremity musculature. Different target areas or affected nerves may have had an impact on performance and outcomes in this study. A control trial would allow a more rigorous comparison of the elements of the intervention.

Participants self-monitored dosage and performed the prescribed intervention independently; therefore, compliance and adherence to the intervention protocol may have had an impact on outcomes. In addition, the variability of activities performed within SES plus activity is a limitation as it was not uniform across all subjects. Other limitations of this study include the small sample size, lack of a control group, and lack of formal measures of adherence to the SES plus activity program. While the results are preliminary, SES plus activity has the potential for clinical benefit and should be further studied in a large, randomized controlled study that includes a feasibility assessment.

## Clinical Implications and Conclusions

We believe that this is the first study that combines SES with standing and mobility activities in a community setting. In an era with limited sources for formal physical therapy, a home base program that produces beneficial functional outcomes is an attractive therapeutic alternative.

Sensory data is related to both physical performance and perceived physical performance. Future studies may consider stratifying research participants based on baseline functional and chronicity level (how long ago they had the stroke). This may inform patient characteristics most associated with change. Establishing evidence based practice guidelines to determine the appropriate dosing of SES parameters to improve LE motor function had remained a challenge.

## Ethics Statement

This study was approved by the Northwestern University Institutional Review Board Office, Northwestern University, Chicago IL (#STU00097364).

## Author's Note

In our study, adults post-stroke wore a sock electrode, which allowed for concurrent delivery of Sensory Electrical Stimulation (SES) during standing and mobility activities. Our findings suggest that this intervention resulted in a significant improvement in the gait speed of younger individuals with a more recent stroke. We believe that this is the first study that combines SES with standing and mobility activities in a community setting.

## Author Contributions

RL-R was designated as first author, wrote, edited, and prepared the manuscript for publication. JS conceived the study and participated in its design and coordination and helped to draft the manuscript. RL-R, JS, MB, and AK participated in data collection and analysis. The authors of this study declared no potential conflicts of interest regarding the research conducted, authorship, or publication of this manuscript.

### Conflict of Interest Statement

The authors declare that the research was conducted in the absence of any commercial or financial relationships that could be construed as a potential conflict of interest. The handling editor is currently editing a Research Topic with one of the authors JS, and confirms the absence of any other collaboration.
